# Supplementary material for: A Likelihood Approach for Real-Time Calibration of Stochastic Compartmental Epidemic Models
Source: PLoS Comput Biol. 2017 Jan 17;13(1):e1005257. doi: 10.1371/journal.pcbi.1005257 (PMC5240920; doi:10.1371/journal.pcbi.1005257)
Supplement: S1 File — (TAR.GZ) [file pcbi.1005257.s014.tar.gz › HSPH_Online-SI-Revision/EnKF07/n1-mild/n1-mild_EnKF_table8.pdf]

1	R0	{1.27677, 7.89375}	{2.81604, 8.71235}	{{2.38646, 3.65649}, {3., 10.0969}}	{120.559, 35.2454}	inf	4.	60.4073	{9.6169, 87.3006}	1412.36	0.926222	newly	2.	53.63	{6., 112.}	2583.5	1.128	Reff	0.856713	0.777524	{0.218182, 2.38407}	63.5376	ar	400.	1767.1	{1501., 2464.}	341.775
2	R0	{1.33231, 10.5259}	{2.84787, 6.49867}	{{2.57019, 3.4599}, {3., 12.2825}}	{113.755, 50.9054}	inf	6.	64.8874	{9.18142, 85.1097}	986.626	0.774816	newly	4.	111.41	{8., 299.}	2691.25	0.939676	Reff	0.714118	0.712676	{0.182149, 2.3289}	72.2493	ar	467.	1712.9	{1442., 2349.}	266.788
3	R0	{1.2273, 6.76944}	{2.56048, 6.29518}	{{1.27083, 3.30216}, {3., 7.80813}}	{108.678, 25.249}	inf	9.	74.0434	{11.0984, 163.68}	728.735	0.716167	newly	6.	82.5	{10., 137.}	1280.67	0.82268	Reff	0.857883	0.489314	{0.0819237, 0.828131}	63.9314	ar	314.	1747.27	{1564., 2168.}	456.455
4	R0	{1.41215, 17.5742}	{2.71223, 6.73003}	{{1.91489, 0.62805}, {3., 14.7051}}	{92.0639, 61.705}	inf	25.	61.6914	{12.6596, 68.9433}	172.526	0.343375	newly	8.	66.24	{9., 74.}	732.5	0.616742	Reff	1.09159	0.420461	{0.104279, 0.662915}	66.0777	ar	465.	1745.49	{1591., 1949.}	275.374
5	R0	{1.42222, 6.15886}	{2.64202, 6.5683}	{{0.719824, 0.952121}, {3., 8.91593}}	{100.874, 38.2185}	inf	11.	43.3594	{6.02621, 41.8513}	312.673	0.439033	newly	5.	40.2	{5., 40.}	712.8	0.613131	Reff	0.742399	0.359506	{0.0657411, 0.541875}	60.3708	ar	490.	1614.42	{1490., 1772.}	229.473
6	R0	{1.24363, 9.95596}	{2.49117, 7.35956}	{{1.20755, 4.57425}, {3., 11.7058}}	{101.589, 35.3563}	inf	11.	43.0912	{10.3617, 36.8336}	301.837	0.425372	newly	12.	38.28	{7., 33.}	242.333	0.344589	Reff	0.863079	0.364431	{0.0542248, 0.539798}	66.0573	ar	411.	1659.89	{1542., 1822.}	303.866
7	R0	{1.36478, 5.96056}	{2.02483, 6.80996}	{{0.164251, 0.653296}, {3., 13.2462}}	{101.322, 87.7937}	inf	8.	76.2995	{6.73967, 110.71}	866.667	0.699233	newly	5.	52.39	{4., 90.}	957.8	0.800686	Reff	0.754723	0.496348	{0.00880109, 1.41443}	78.1103	ar	484.	1829.76	{1614., 2431.}	278.05
8	R0	{1.36335, 17.6429}	{2.69362, 5.44823}	{{1.80719, 0.6672}, {3., 10.1045}}	{97.5735, 69.1194}	inf	28.	54.7216	{6.40104, 76.146}	141.268	0.36494	newly	12.	106.01	{6., 174.}	804.25	0.567184	Reff	1.02251	0.644652	{0.206937, 1.24681}	54.693	ar	484.	1599.31	{1411., 1960.}	230.436
9	R0	{1.26575, 5.28707}	{2.69111, 4.92689}	{{2.09618, 1.77805}, {3., 12.6547}}	{112.61, 78.0899}	inf	6.	55.6013	{3.54399, 49.862}	844.179	0.69627	newly	5.	75.97	{4., 76.}	1432.2	0.747171	Reff	0.669582	0.34161	{0.0279073, 0.599805}	69.9369	ar	472.	1597.67	{1437., 1845.}	238.489
10	R0	{1.42942, 9.49905}	{2.80951, 10.3248}	{{2.3855, 3.23036}, {3., 16.7282}}	{96.5487, 60.1815}	inf	1.	55.0111	{7.96265, 58.1178}	5401.11	1.52054	newly	1.	31.31	{7., 53.}	3033.	1.32166	Reff	0.760451	0.500897	{0.0761856, 0.831606}	67.8816	ar	468.	1666.69	{1500., 2006.}	256.13
11	R0	{1.46827, 15.0983}	{2.31712, 4.94963}	{{0.91446, 1.33996}, {3., 10.4011}}	{81.9784, 67.2173}	inf	22.	70.6412	{6.28245, 123.673}	246.917	0.446054	newly	11.	86.91	{6., 128.}	703.545	0.61432	Reff	1.12469	0.361346	{0.0966304, 0.596226}	69.6206	ar	495.	1679.73	{1518., 2075.}	239.339
12	R0	{1.25751, 13.279}	{2.72619, 5.75271}	{{2.07752, 0.526316}, {3., 10.0894}}	{116.792, 61.2076}	inf	16.	67.7384	{12.019, 84.9948}	337.107	0.463096	newly	9.	88.46	{7., 133.}	894.	0.672224	Reff	0.975828	0.556901	{0.212738, 0.764933}	61.7723	ar	366.	1644.29	{1466., 2279.}	349.26
13	R0	{1.3425, 9.16361}	{2.87565, 4.19875}	{{2.40787, 1.52895}, {3., 7.59318}}	{114.201, 55.2626}	inf	2.	25.5175	{3.52224, 28.5413}	1181.15	0.820696	newly	0.1	41.39	{4., 66.}	41294.	2.14393	Reff	0.753143	0.440956	{0.236898, 0.588071}	57.0442	ar	442.	1562.24	{1456., 1742.}	253.448
14	R0	{1.18936, 6.20583}	{2.70029, 3.75886}	{{0.724351, 1.05479}, {3., 7.84979}}	{136.5, 52.353}	inf	3.	32.3027	{5.74245, 35.4264}	980.495	0.841382	newly	3.	42.3	{6., 48.}	1312.67	0.874576	Reff	0.735024	0.38119	{0.0781457, 0.514941}	59.665	ar	382.	1600.87	{1510., 1714.}	319.076
15	R0	{1.27137, 9.78868}	{2.41982, 2.68399}	{{0.608855, 0.672232}, {3., 3.3845}}	{115.053, 74.2056}	inf	16.	54.3149	{7.75883, 78.1014}	265.389	0.363789	newly	13.	67.04	{8., 94.}	432.308	0.454303	Reff	0.752651	0.274397	{0.0105248, 0.59192}	64.5316	ar	496.	1661.82	{1521., 1899.}	235.044
16	R0	{1.23102, 13.6452}	{2.71344, 5.39192}	{{2.02677, 3.41265}, {3., 7.37187}}	{120.422, 60.4849}	inf	26.	17.2785	{3.20447, 17.7788}	80.9364	0.521549	newly	13.	29.46	{2., 24.}	200.615	0.442536	Reff	0.853097	0.412654	{0.131054, 0.692967}	65.5135	ar	445.	1566.22	{1470., 1711.}	251.96
17	R0	{1.27213, 13.7452}	{2.49928, 3.87568}	{{1.4419, 1.4087}, {3., 5.70934}}	{98.2599, 72.3662}	inf	30.	15.2079	{1.84235, 26.4977}	86.1809	0.691601	newly	16.	36.5	{2., 45.}	221.375	0.554355	Reff	0.957914	0.482322	{0.119487, 0.974565}	56.7654	ar	367.	1584.66	{1490., 1843.}	331.787
18	R0	{1.25318, 11.2151}	{2.45836, 4.34213}	{{0.904349, 2.50351}, {3., 10.2789}}	{110.129, 61.2832}	inf	24.	58.0659	{11.8914, 90.6858}	163.449	0.387323	newly	17.	67.34	{8., 142.}	315.059	0.5285	Reff	0.833365	0.404635	{0.0490528, 0.782491}	58.7324	ar	416.	1544.52	{1439., 1704.}	271.279
19	R0	{1.46006, 8.11496}	{2.5142, 8.0285}	{{1.59022, 3.64664}, {3., 11.7959}}	{74.354, 24.2658}	inf	20.	74.3428	{11.1368, 121.937}	289.41	0.463904	newly	12.	79.9	{8., 170.}	581.167	0.565804	Reff	0.762151	0.65769	{0.146352, 1.47435}	61.7849	ar	496.	1772.34	{1539., 2312.}	257.327
20	R0	{1.14273, 6.71261}	{2.77087, 4.16052}	{{2.18019, 0.486271}, {3., 9.31567}}	{142.478, 51.6675}	inf	15.	46.6385	{4.53484, 61.2841}	239.612	0.384222	newly	11.	62.14	{5., 95.}	492.545	0.535689	Reff	0.742775	0.462344	{0.132694, 0.863031}	59.3564	ar	362.	1702.96	{1546., 2173.}	370.431
21	R0	{1.42432, 8.23905}	{2.77352, 11.3275}	{{1.92205, 3.17022}, {3., 18.5476}}	{94.7262, 59.7589}	inf	6.	75.1551	{29.7114, 110.792}	1152.58	0.997392	newly	5.	55.56	{13., 112.}	1011.6	0.844479	Reff	0.853168	0.53194	{0.296856, 0.686172}	52.112	ar	405.	1677.64	{1526., 1804.}	314.232
22	R0	{1.29386, 17.863}	{2.25679, 2.44579}	{{0.544272, 1.02447}, {3., 5.37953}}	{107.086, 86.308}	inf	35.	50.4423	{4.91083, 66.4876}	101.176	0.335098	newly	16.	79.63	{6., 100.}	418.438	0.501969	Reff	0.98204	0.428913	{0.0261967, 0.619975}	69.9042	ar	488.	1631.64	{1492., 1733.}	234.352
23	R0	{1.39274, 13.0167}	{2.74752, 5.53564}	{{2.02286, 0.993447}, {3., 19.4262}}	{97.2745, 71.6307}	inf	19.	47.7195	{8.09648, 52.4251}	175.404	0.276082	newly	8.	44.52	{6., 87.}	466.5	0.579791	Reff	0.799433	0.529475	{0.11599, 1.18803}	66.1681	ar	475.	1570.11	{1399., 2080.}	230.549
24	R0	{1.32178, 15.8991}	{2.39777, 5.28086}	{{1.33017, 0.104761}, {3., 11.097}}	{89.7921, 66.7852}	inf	28.	59.767	{5.91668, 111.571}	152.523	0.32677	newly	13.	73.33	{4., 166.}	487.615	0.522133	Reff	0.810251	0.556053	{0.107684, 1.36787}	57.0624	ar	471.	1681.86	{1480., 2121.}	257.083
25	R0	{1.37568, 17.8427}	{2.6435, 2.97466}	{{1.76303, 1.14971}, {3., 5.67605}}	{92.1592, 83.4052}	inf	24.	57.6684	{5.28438, 75.7375}	182.623	0.393555	newly	8.	75.18	{6., 91.}	854.5	0.675363	Reff	1.00837	0.329577	{0.157173, 0.548208}	68.568	ar	398.	1514.85	{1396., 1555.}	280.616
26	R0	{1.20993, 6.4656}	{2.51736, 5.1302}	{{1.47582, 0.0624538}, {3., 14.4313}}	{112.627, 54.5419}	inf	2.	78.9896	{7.50985, 209.507}	3850.31	1.26393	newly	1.	82.59	{6., 209.}	8159.	1.52842	Reff	0.803394	0.447775	{0.208429, 0.537976}	56.3245	ar	345.	1688.58	{1493., 2239.}	389.443
27	R0	{1.20548, 13.8003}	{2.49777, 6.25831}	{{0.886759, 0.35554}, {3., 13.2534}}	{119.071, 55.1233}	inf	23.	46.0403	{4.52701, 75.8826}	151.163	0.351369	newly	10.	39.78	{4., 63.}	324.6	0.43714	Reff	0.817315	0.426884	{0.0930643, 0.899954}	70.9212	ar	350.	1542.6	{1416., 1776.}	340.743
28	R0	{1.3176, 14.6594}	{2.49913, 5.15146}	{{0.301708, 0.268238}, {3., 9.93686}}	{117.803, 64.859}	inf	16.	48.4538	{5.024, 86.62}	234.604	0.364201	newly	8.	52.81	{4., 138.}	580.125	0.595103	Reff	1.06989	0.625134	{0.021726, 1.77697}	73.1653	ar	362.	1701.52	{1517., 2372.}	370.033
29	R0	{1.28202, 6.23047}	{2.3279, 3.83843}	{{0.682406, 3.36551}, {3., 4.33079}}	{108.326, 38.3926}	inf	4.	55.5293	{5.29773, 153.21}	1296.71	0.804228	newly	5.	76.81	{5., 172.}	1445.4	0.782533	Reff	0.782032	0.465381	{0.0994524, 0.573107}	53.8453	ar	391.	1623.43	{1465., 2079.}	315.199
30	R0	{1.28177, 8.83252}	{2.6362, 6.02294}	{{1.93046, 3.25663}, {3., 11.9998}}	{105.669, 49.4713}	inf	18.	48.6813	{5.00266, 60.3374}	204.467	0.427373	newly	11.	62.07	{5., 99.}	485.727	0.530248	Reff	0.76778	0.541998	{0.157211, 1.0827}	60.735	ar	427.	1711.84	{1531., 2281.}	300.899
31	R0	{1.35886, 5.97708}	{2.81355, 5.85941}	{{2.36359, 2.52979}, {3., 7.90332}}	{107.052, 47.5699}	inf	0.1	64.7854	{7.66081, 75.6097}	64686.4	2.51287	newly	0.1	64.51	{7., 91.}	64414.	2.49706	Reff	0.69166	0.563212	{0.144194, 0.626582}	46.3939	ar	491.	1750.56	{1596., 1982.}	256.53
32	R0	{1.27601, 17.9804}	{2.28496, 4.77935}	{{0.600792, 2.56734}, {3., 8.2964}}	{110.08, 73.4909}	inf	36.	82.4787	{5.46221, 310.897}	174.721	0.374302	newly	15.	72.99	{5., 240.}	411.533	0.543236	Reff	0.962112	0.481391	{0.106283, 0.692502}	54.598	ar	445.	1625.22	{1447., 2155.}	265.218
33	R0	{1.3368, 11.3235}	{2.75459, 8.5788}	{{2.23232, 3.24783}, {3., 11.5023}}	{108.296, 34.2386}	inf	13.	114.882	{7.02699, 255.265}	799.681	0.658875	newly	7.	114.95	{7., 394.}	1555.57	0.776312	Reff	0.788712	0.655085	{0.257293, 1.37266}	54.5909	ar	417.	1813.77	{1511., 2526.}	334.957
34	R0	{1.21021, 11.3635}	{2.51614, 10.3689}	{{1.63897, 1.39323}, {3., 18.508}}	{113.397, 46.5926}	inf	18.	123.909	{5.20484, 507.657}	619.213	0.571499	newly	15.	64.35	{2., 280.}	375.267	0.555156	Reff	0.905237	0.451295	{0.105868, 0.802081}	68.7041	ar	327.	1778.82	{1496., 2556.}	443.982
35	R0	{1.53382, 10.8866}	{2.84288, 9.97602}	{{2.33619, 3.42834}, {3., 15.4045}}	{85.3466, 47.3749}	inf	21.	58.0291	{6.42337, 79.9446}	196.371	0.426021	newly	15.	58.07	{4., 154.}	313.4	0.48091	Reff	0.852804	0.628508	{0.165327, 1.40535}	64.3526	ar	476.	1708.36	{1509., 2357.}	258.899
36	R0	{1.20423, 6.83844}	{2.55606, 7.18323}	{{1.27848, 0.349175}, {3., 11.2546}}	{113.881, 46.5074}	inf	0.1	90.0125	{9.4428, 188.03}	89912.5	2.61603	newly	0.1	108.93	{7., 266.}	108832.	2.53957	Reff	0.70327	0.645385	{0.143431, 1.38683}	66.5824	ar	416.	1831.42	{1565., 2498.}	340.245
37	R0	{1.43629, 10.1548}	{2.70167, 6.56027}	{{1.84938, 3.14631}, {3., 10.4292}}	{88.1004, 39.2118}	inf	12.	65.6774	{16.7285, 102.526}	454.891	0.598462	newly	8.	63.9	{10., 191.}	706.5	0.720978	Reff	0.963751	0.516912	{0.165069, 0.761942}	71.0725	ar	367.	1678.52	{1475., 2349.}	357.362
38	R0	{1.37504, 8.93428}	{2.65947, 5.5892}	{{2.04457, 0.864241}, {3., 7.67631}}	{95.4005, 50.1708}	inf	7.	60.0932	{10.5852, 69.2146}	765.046	0.731718	newly	5.	79.08	{9., 219.}	1488.8	0.884234	Reff	0.717771	0.508055	{0.0741384, 1.11948}	69.769	ar	483.	1704.81	{1520., 2205.}	252.963
39	R0	{1.51637, 9.36742}	{2.68266, 11.5847}	{{1.81212, 5.91114}, {3., 15.0696}}	{79.0407, 34.8101}	inf	9.	64.1794	{16.2495, 74.0099}	616.884	0.72568	newly	4.	38.06	{9., 53.}	856.	0.818826	Reff	0.884044	0.600249	{0.0763334, 1.02572}	56.7366	ar	441.	1720.16	{1530., 1963.}	290.059
40	R0	{1.23658, 9.82869}	{2.70598, 2.589}	{{2.08746, 1.33014}, {3., 4.30599}}	{118.828, 73.6587}	inf	1.	54.0151	{2.54411, 95.5005}	5309.	1.29119	newly	1.	95.95	{3., 342.}	9505.	1.4605	Reff	0.724636	0.532028	{0.163061, 1.22558}	61.7833	ar	414.	1760.46	{1559., 2147.}	325.232
41	R0	{1.24247, 5.4242}	{2.53606, 8.78768}	{{0.493234, 3.58047}, {3., 12.5218}}	{124.467, 73.2552}	inf	5.	55.6635	{4.61671, 50.6482}	1022.23	0.720754	newly	5.	35.02	{3., 42.}	616.4	0.615615	Reff	0.821273	0.476992	{0.0654877, 0.764126}	63.9705	ar	345.	1691.87	{1545., 1998.}	390.397
42	R0	{1.28208, 4.40223}	{2.78548, 6.23532}	{{2.42046, 2.14606}, {3., 12.2201}}	{118.675, 68.3684}	inf	0.1	85.5352	{15.4401, 94.6719}	85435.2	2.64953	newly	0.1	95.39	{10., 225.}	95290.	2.61194	Reff	0.847455	0.526491	{0.0550416, 0.870407}	64.4692	ar	339.	1785.41	{1580., 2221.}	426.67
43	R0	{1.56773, 6.50474}	{2.56384, 5.43622}	{{1.24138, 0.567111}, {3., 10.5499}}	{73.4998, 45.0278}	inf	3.	59.1805	{8.77358, 61.5973}	1872.77	0.987026	newly	2.	94.36	{6., 73.}	4618.	1.16502	Reff	0.790136	0.528117	{0.184481, 0.869422}	53.1262	ar	498.	1765.07	{1595., 2075.}	254.432
44	R0	{1.10489, 6.01941}	{2.6605, 5.30465}	{{1.30873, 3.25335}, {3., 6.5679}}	{140.794, 31.2467}	inf	1.	41.1649	{5.43709, 53.312}	4017.15	1.37043	newly	0.1	40.42	{4., 71.}	40324.	2.28839	Reff	0.724808	0.389045	{0.101784, 0.561589}	61.5212	ar	346.	1595.23	{1472., 1818.}	361.049
45	R0	{1.25888, 14.1926}	{2.37625, 6.52069}	{{1.08919, 0.11127}, {3., 16.4393}}	{96.5726, 61.4782}	inf	17.	61.9908	{11.8039, 93.4485}	277.388	0.465006	newly	7.	58.02	{12., 93.}	733.143	0.705218	Reff	0.682313	0.416925	{0.123425, 0.782936}	53.9942	ar	471.	1614.13	{1473., 1956.}	242.703
46	R0	{1.45292, 16.616}	{2.52222, 8.95837}	{{1.65156, 2.6991}, {3., 12.1859}}	{77.4864, 46.9862}	inf	28.	43.9786	{12.222, 72.6663}	81.9516	0.273341	newly	9.	35.59	{6., 67.}	309.889	0.520924	Reff	1.03012	0.483316	{0.127846, 0.825867}	61.7778	ar	494.	1625.53	{1493., 1724.}	229.055
47	R0	{1.22528, 16.6033}	{2.78186, 3.86582}	{{2.26867, 0.3221}, {3., 5.54795}}	{128.712, 77.1566}	inf	30.	57.4918	{3.49453, 165.394}	176.811	0.48358	newly	10.	104.89	{4., 349.}	975.3	0.640134	Reff	1.00963	0.698075	{0.0520412, 2.20238}	68.1781	ar	368.	1751.49	{1508., 2422.}	375.948
48	R0	{1.1669, 6.92715}	{2.57548, 5.29456}	{{1.41954, 2.26658}, {3., 10.9158}}	{120.711, 51.3653}	inf	6.	38.3399	{6.72756, 60.7359}	549.088	0.66695	newly	3.	54.71	{5., 88.}	1729.67	0.937453	Reff	0.735147	0.509631	{0.0626351, 1.22106}	68.9652	ar	416.	1617.18	{1481., 1832.}	288.745
49	R0	{1.32749, 16.0887}	{2.61919, 6.15729}	{{1.70051, 2.87461}, {3., 8.08519}}	{97.3039, 61.7291}	inf	18.	109.029	{8.94296, 264.82}	519.189	0.513745	newly	3.	97.65	{8., 296.}	3155.	1.127	Reff	1.06863	0.498904	{0.17391, 0.77778}	61.1696	ar	353.	1764.84	{1477., 2600.}	399.955
50	R0	{1.29636, 15.2609}	{2.68922, 6.74395}	{{1.79129, 3.08403}, {3., 7.72371}}	{107.444, 56.1122}	inf	33.	40.7246	{5.90785, 53.4444}	61.7105	0.285277	newly	13.	34.71	{5., 49.}	192.231	0.465678	Reff	0.735036	0.450587	{0.108782, 0.641896}	55.6093	ar	470.	1551.62	{1402., 1723.}	230.132
